# KISSa: a strategy to build multiple sequence alignments from pairwise comparisons of very closely related sequences

**DOI:** 10.1186/1756-0500-2-91

**Published:** 2009-05-20

**Authors:** Francesco Marass, Chris Upton

**Affiliations:** 1 Biochemistry and Microbiology, University of Victoria, Victoria, BC V8W 3P6, Canada

## Abstract

**Background:**

The volume of viral genomic sequence data continues to increase rapidly. This is especially true for the smaller RNA viruses, which are relatively easy to sequence in large numbers. The data volumes cause a number of significant problems for research applications that require large multiple alignments of essentially complete genomes, which are of the order of 10 kb.

**Findings:**

We present a simple strategy to enable the creation of large quasi-multiple sequence alignments from pairwise alignment data. This process is suitable for large, closely related sequences such as the polyproteins of dengue viruses, which need the insertion of very few indels.

**Conclusion:**

The quasi-multiple sequence alignments generated by KISSa are sufficiently accurate to support tree-based genome selection for interactive bioinformatics analysis tools. The speed of this process is critical to providing an interactive experience for the user.

## Background

There are many reasons for constructing multiple sequence alignments (MSA), which form the backbone of comparative analyses and the starting point of phylogenetic studies. Similarly, there are a variety of algorithms (CLUSTAL [1,2], T-Coffee [3], MUSCLE [4]) and more software tools to allow a researcher to input a series of DNA or protein sequences and obtain an MSA. The output is usually viewed using a graphical user interface (GUI) that may also permit editing of the MSA (Jalview [5,6], Base-By-Base (BBB) [7]). The alignment of large DNA sequences, in the size range of bacterial chromosomes, requires specialized alignment tools and viewers [8,9]. The mandate of the NIH funded Viral Bioinformatics Resource Center (VBRC) includes collection, annotation and storage of the complete genomes for seven virus families, and both the VBRC administrators and researchers frequently create MSAs of genes, proteins and genomes. Recently, the selection of genomes from large data sets, the VBRC current has >1200 dengue virus genomes (4 genotypes), using menu-type lists has become onerous and we are considering graphical tools based on phylogenetic trees to help users through the data selection process. This will require frequent generation of large MSAs and use considerable computation time because, as new genomes are added to the VBRC database, trees need to be regenerated. Although these alignments could be done at off-peak times, or the new sequences added to a pre-existing alignment with MUSCLE [4], VBRC users also frequently want to make specific selections of genomes that might be in the order of several dozen or several hundred in number and perform on-the-fly MSAs.

Dengue viruses belong to the *Flaviviridae *taxonomic family and possess a genome comprised of a single molecule of positive sense single stranded RNA (+ssRNA). The genome is essentially a single Open Reading Frame (ORF) that is translated into a large polyprotein (approximately 3390 amino acids), which is proteolytically cleaved into a series of mature proteins. Therefore, when these polyproteins are aligned, the vast majority of the genomic variation is captured. Furthermore, when we examined the 1273 dengue virus genomes, it was apparent that the vast majority of variation between the genomes was at the 5' and 3' ends of the genomes in untranslated regions (UTR) and resulted from incomplete genome sequencing. When focusing on the viruses belonging to a single genotype, we realized that the variation among the polyproteins was almost totally restricted to amino acid substitutions with very few occurrences of insertions or deletions (INDELS). Although the presence of at least some INDELS leads to a requirement for some sort of alignment process, we questioned whether a full MSA needed to be performed. Specifically, we decided to test whether pairwise alignments of each sequence to a *reference *sequence would be sufficient to build a *quasi*-MSA that could function for our purposes. Also, since we already store the genome and polyprotein sequences in the VBRC database, we wanted to create a shorthand representation (tags) for the alignment of each sequence to a *reference *that could be stored and used to build *ad hoc *MSAs as required by a VBRC user.

The dengue virus polyproteins were chosen as a test case because these contain the complete viral protein-coding information and exclude the variation, and possible errors, in the viral 5' and 3' UTRs due to incomplete sequencing of the viral genomes. These large proteins of approximately 3400 amino acids contain sufficient information to build the trees required for navigating sets of genomes selected by VBRC users.

**Figure 1:**
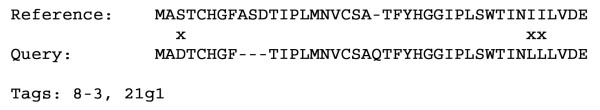
**Use of tags to create an alignment**. To create this alignment, the query sequence read against the reference sequence until after amino acid #8 (reference sequence numbering), then a gap of 3 residues is inserted in the query (tag 8-3; deletion of length 3 in query). The sequences are continued until amino acid #21 (reference sequence numbering), then a gap of 1 residue is the reference sequence (tag 21g1; insertion of 1 amino acid in query sequence). There is no need for substitution tags in this example because both amino acid sequences have been stored.

The goal of this paper is to show the utility of encoding and storing pairwise alignment data as shorthand tags and that quasi-MSAs can be very quickly built with this information. KISSa (**K**eep **I**t **S**imple **S**equence **a**lignment) is composed of two Perl scripts that: 1) the generation of alignment tags (Figure 1) through pairwise alignments (PA), and 2) the construction of a quasi multiple sequence alignment (MSA) from the tags. The Bioperl toolkit was utilized [10].

**Figure 2:**
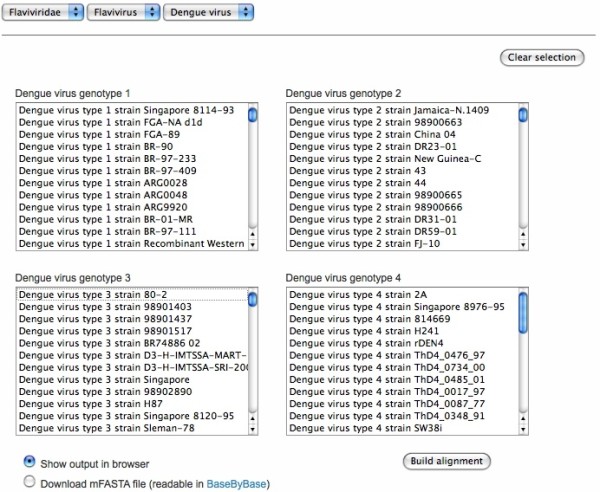
**Web interface for the demonstration of KISSa**. Four menus are used to select polyproteins from different dengue virus genotypes. Results can be viewed in a browser window or exported in mFASTA format for loading into an MSA viewer/editor such as Base-By-Base.

In our current demonstration of KISSa, we use web-forms to display menus of all dengue genomes in the VBRC database (Figure 2). Nightly, scripts check for new genomes added to the database, run pairwise alignments and create and store the shorthand tags in text files.

### Generation of alignment tags

Since the sequences in the VBRC database are named by genotype, for KISSa we chose a reference sequence for each genotype set against which all sequences in the set are aligned in a pairwise fashion. PAs are computed using NEEDLE, the EMBOSS [11] implementation of the Needleman-Wunsch algorithm [12]. The PA output is parsed to generate alignment tags, which are specific to a reference-sequence alignment and recreate the alignment when applied to their sequence. Whenever a gap is encountered in the PA output, a corresponding tag is created. Tags mark deletions (if the gap is in the reference sequence) and insertions (if the gap is in the query sequence). The alignment tags are stored and used later in the construction of MSAs.

Tags have a simple syntax and consist of three fields: location, action, and additional data (Table 1). The location is the position of a difference between an aligned reference-sequence pair (Figure 1). The position is relative to the reference sequence to ensure that locations are valid for all sequences in the set, because all sequences are aligned against the same reference. Action is the type of difference: insertion, deletion, or substitution. Additional data are the object of the difference: in case of an insertion, the datum is the nucleotides or amino acids that need to be inserted; for deletions it is the length of gap.

**Table 1:** Examples of KISSa tags.

Tag	Consequence
3-1	Deletion of length 1, starting at position 3 in the reference sequence
17sT	Replace nucleotide at position 17 in the reference sequence with T
5+ME	Insert two amino acids (ME) starting at position 5 in the reference sequence
6g3	Insert a gap of length 3 at position 6
0-0	The no-operation tag, a deletion of zero characters

An alternative method, more suited to a stand-alone application, but slightly slower, is to also store substitutions between the reference and query sequences. In this case, since the query sequence need not be stored, very large numbers of sequences can be stored as alignment tags together with their reference in a small footprint if the sequences are very similar. However, in this case the amino acid sequences would also be reconstructed from the reference and tags before beginning the alignment.

### Construction of MSA

To construct a quasi-MSA, KISSa uses a list of sequences, their alignment tags and the reference sequence. The alignment is computed in a series of steps by applying the alignment tags of the sequences the user wishes to align. Deletion rules are applied first, because they affect only the sequence to which they belong; insertion rules are applied afterwards, as they affect all other sequences (if a sequence has an insertion, the reference and other sequences require the insertion of a gap), and need additional processing to take care of that complications arising from overlap of insertions. To distinguish between processed and non-processed insertion tags, a new tag, called a gap rule, is used. These have the same location as the insertion rules from which they are generated, but rather than specifying the nucleotides or amino acids to be inserted, they specify the length of the gap that needs to be inserted in all other sequences. For example, in an MSA two or more sequences might share an insertion, meaning that, relative to the reference, all these sequences have at the same position an insertion of the same length. In this case, they all produce the same gap rule, thus only one gap has to be inserted to ensure the alignment.

Adjustments are also needed to compensate for overlapping insertions. In this case, the gap rules corresponding to each of the overlapping insertions are merged together and the composite gap rule starts at the first location of the overlapping insertions, and ends at the last location. When applied, sequences with an insertion included by this rule will be padded with a gap as large as the difference between the length of the gap rule and the length of the insertion of the sequence. However, it is possible that overlapping insertions come from the same sequence due to the indexing of locations relative to the reference sequence. If these rules were merged the alignment would be broken, therefore, gap rules are also tagged with the ID of each sequence from which they arise. If two gap rules have at least one ID in common, they are not merged. Finally, since the location of the alignment tags is relative to the reference, and insertions and gaps change the indexing of the sequence, we use an offset to track the shifts in locations.

### Intergenotype MSA

Obviously, there are more differences between dengue viruses from different genotypes, however, we found that KISSa can also be used to create inter-genotypic quasi-MSAs for these viruses. Inter-genotypic, or multiset, alignments require that the references, one for each set, be in turn aligned to a common reference, called the *master reference*, whichcan be one of the original reference sequences. The construction of an inter-genotypic MSA has to account for the alignment tags of the references, therefore, the deletion rules have to be applied to all sequences in the set of the reference, while the non-processed insertion rules are applied to all sequences that do not belong to the sets of the reference. The reference alignment tags are applied between the sequence deletion tags and gap rules.

### Complexity and Performance

KISSa is composed of two separate steps: the creation of alignment tags from PAs, and the use of the alignment tags to build a quasi-MSA. The first step relies on NEEDLE to perform *n *PAs. Space and time complexities for NEEDLE are O(*l*^2^), where *l *is the length of the longest sequence. The PA output is then parsed in O(*l*) using O(*nd*) space, where *d *is the maximum number of alignment tags per sequence. Because the sequences are closely related, *d *is usually orders of magnitude smaller than *l*. The overall time complexity for the generation of the alignment tags is O(*nl*^2^).

The generation of the quasi-MSA has O(*nl*) + O(*nd*) = O(*nl*) space complexity, since that is the space needed to store *n *sequences of length *l *(the output) and their alignment tags. The worst case time complexity for this step is O(*nd*^3^*l*), of which O(*ndl*) to apply deletion tags and gather gap rules, O(*d*^2^) to merge the gap rules, and O(*nd*^3^*l*) to apply them.

The generation of alignment tags is the longer step, but it only has to happen once. Afterwards, for the creation of quasi MSA, KISSa performs in O(*nd*^3^*l*), which is linear time with respect to the number of sequences being aligned, but polynomial with respect to the number of indels, which is small when dealing with closely related viruses. Performance data is presented in Table 2. Building the KISSa quasi-MSA of all dengue virus poly proteins took less than 30 sec whereas use of MUSCLE with parameters (-maxiters 1 -diags -sv -distance1 kbit20_3) for fastest possible protein alignment took 43 minutes on a desktop machine. Both the speed of KISSa and the ability to review the results quickly in a web browser window

**Table 2:** Example KISSa alignments. Number of differences refers to the total number of insertions and deletions in all sequences.

Genotypes	# of Seqs	Time for KISSa MSA (secs)	Number of differences
DENV 1	528	3.970	8 deletions, 4 insertions
DENV 2	558	0.284	3 deletions
DENV 3	236	0.697	1 insertion
DENV 4	31	0.164	3 deletions
DENV 1,2	1086	10.607	1127 deletions, 562 insertions
DENV 1,2,3	1322	20.289	1599 deletions, 1035 insertions
DENV 1,2,3,4	1354	25.022	1755 deletions, 1129 insertions

### Limitations

Clearly, the limitation of KISSa is its dependency on the use of sequences that are highly similar, although it does function with different genotypes of a virus species such as dengue virus. KISSa should not be viewed as a multiple sequence alignment algorithm such as MUSCLE, but rather as a tool to format sequences that have 1) minor differences in length and 2) very few indels into a quasi-MSA. The position of gaps is decided by the Needleman-Wunsch pairwise alignment process. However, there are a variety of instances in which very rapid, essentially interactive, alignment building is of great value. For example, when selecting a large number of genomes from a database for alignment it may not be possible for the user to screen out truncated genomes or genomes that contain unusual, perhaps erroneous, INDELS. This post-alignment review, re-selection of sequences and re-alignment can be very slow if standard alignment tools are used. However, with KISSa, the user can very rapidly follow this multi-stage process to arrive at the required alignment. In this situation, the ability to quickly view the KISSa alignment in a web browser window, rather than import into an MSA editor, allows a user to rapidly identify sequences that should be omitted from the next round of alignment.

## Conclusion

We presented a method for quickly building useful alignments of a large number of closely related sequences (DNA or protein). The alignments are constructed from alignment tags, generated from pairwise alignments of query sequences against a common reference. In our example using dengue virus polyproteins, we were able to generate MSAs with polyproteins from different dengue virus genotypes that were sufficient for constructing a phylogenetic tree for input into in a graphical interface for genome selection. Since most phyogenetic tree construction ignores MSA columns with gaps, minor imperfections are not a great consequence for this use.

Obviously, the KISSa constructed alignment protocol will be less reliable when multiple gaps are required in small regions, a true MSA algorithm is needed to score and optimize these regions. However, some software (e.g. BBB) allows small regions of a large MSA with very long sequences to be re-aligned with algorithms such as MUSCLE or T-coffee; BBB also provides visual feedback of differences between adjacent sequences, so it is very easy to spot regions that a user may wish to inspect.

## Availability and requirements

**Project name: **Keep It Simple Sequence alignment (KISSa)

**Project homepage: **

**Operating system: **Platform independent

**Programming language: **Perl (Bioperl)

**Other requirements: **None

**License: **KISSa scripts are distributed under the Open Software License.

**Any restrictions to use by non-academics: **None

## Competing interests

The authors declare that they have no competing interests.

## Authors' contributions

CU conceived the idea and specifications; FM developed the code; both authors tested the tool and contributed to writing the manuscript. Both authors read and approved the final manuscript.
